# Dynamic photo-switching in light-responsive JUC-62 for CO_**2**_**capture**

**DOI:** 10.1038/s41598-017-13536-4

**Published:** 2017-10-17

**Authors:** Nicholaus Prasetya, Bradley P. Ladewig

**Affiliations:** Barrer Centre, Department of Chemical Engineering, Imperial College London, South Kensington, SW7 2AZ United Kingdom

## Abstract

In this paper, we demonstrate the highly efficient photo-switching ability of a Cu-azobenzene tetracarboxylate MOF (JUC-62) for low-energy CO_2_ capture. Under UV light irradiation, both at 273 and 298 K, JUC-62 showed 51% and 34% lower CO_2_ uptake, respectively, than when UV light was off. Its dynamic CO_2_ uptake also matched well with its static condition. Storing it at ambient condition was also found not to destroy its framework structure and its dynamic photoswitching property could still be maintained.

## Introduction

Metal-organic framework (MOF) is a hybrid structure built by inorganic metal and organic linkers. MOFs gain increased interest because of their high surface area that render them suitable as future adsorbents for various applications. Recently, there is an increased interest in synthesizing stimuli-responsive MOFs that have different behaviour upon the exposure to external stimuli such as temperature, pH and light. Among the stimulants, light can be considered as the most convenient thanks to its abundance. Such MOFs are then usually called light-responsive MOFs.

Light-responsive MOFs have significant potential to be applied as advanced adsorbent for CO_2_ capture. This is because such MOFs have different CO_2_ uptake in the presence or absence of light with a particular wavelength such as 365 nm UV or visible light. Therefore, this MOF can then be exploited in a light-induced swing adsorption (LISA) process^1,2^. The advantage of using LISA compared with conventional adsorption processes is realised during the regeneration process. Regeneration of light-responsive MOFs can be completed by simply irradiating the light-responsive MOF with light at a certain wavelength^1^. It then eliminates the requirement of temperature or pressure change as used in conventional processes^3^.

Up to now, many different types of light-responsive MOFs have been successfully synthesized. In general, they can be categorized into three generations. Generation-1 light-responsive MOF utilizes light-responsive guest molecule to induce its light-responsive property. The example for this MOFs have been given by Yanai *et al*.^4^ and Lyndon *et al*. who utilized visible light to trigger CO_2_ release^5^.

In contrast to generation-1, generation-2 and generation-3 light-responsive MOFs uses light-responsive ligands to induce a light-responsive property^6^. In generation-2, the light-responsive moiety protrudes into the MOF’s pore. One of the famous examples of this MOF was given by Zhou and co-workers by synthesizing a light-responsive isoreticular MOF-5^7^. The disadvantage of using generation-1 and generation-2 light-responsive MOF resides on the fact that their pores are occupied either by guest molecules or other moieties. This will potentially reduce their effectiveness when being used as adsorbent.

Meanwhile in generation-3, the light-responsive ligand acts as a pillaring framework of the MOF. There is one obvious advantage of utilizing generation-3 light responsive MOF: the absence of any molecules inside its pore. This renders the MOF’s pore to be fully accessible once they are going to be used as an adsorbent. Various generation-3 light-responsive MOFs have then been investigated this far such as Zn–(AzDC)(4,4′-BPE)_0.5_ MOF^8^ and PCN-250^1^.

We then chose to enrich the candidates for generation-3 light-responsive MOF. In this regard, we chose JUC-62^9^ as another candidate for generation-3 light-responsive MOF for CO_2_ capture. Previous investigations have shown the applicability of this particular MOF for hydrogen storage application^9^ and metal adsorbent^10^. However, its CO_2_ adsorption capacity and in particular, its potentiality as a light-responsive adsorbent have not been investigated. We are then interested to explore the applicability of this particular MOF since it bears light-responsive pillar ligand that might enable it to have a photo-switching ability. Apart from that, JUC-62 is also relatively stable at ambient condition thanks to its interpenetrated and catenated structure and thus makes them more applicable.

## Results

**Figure 1:**
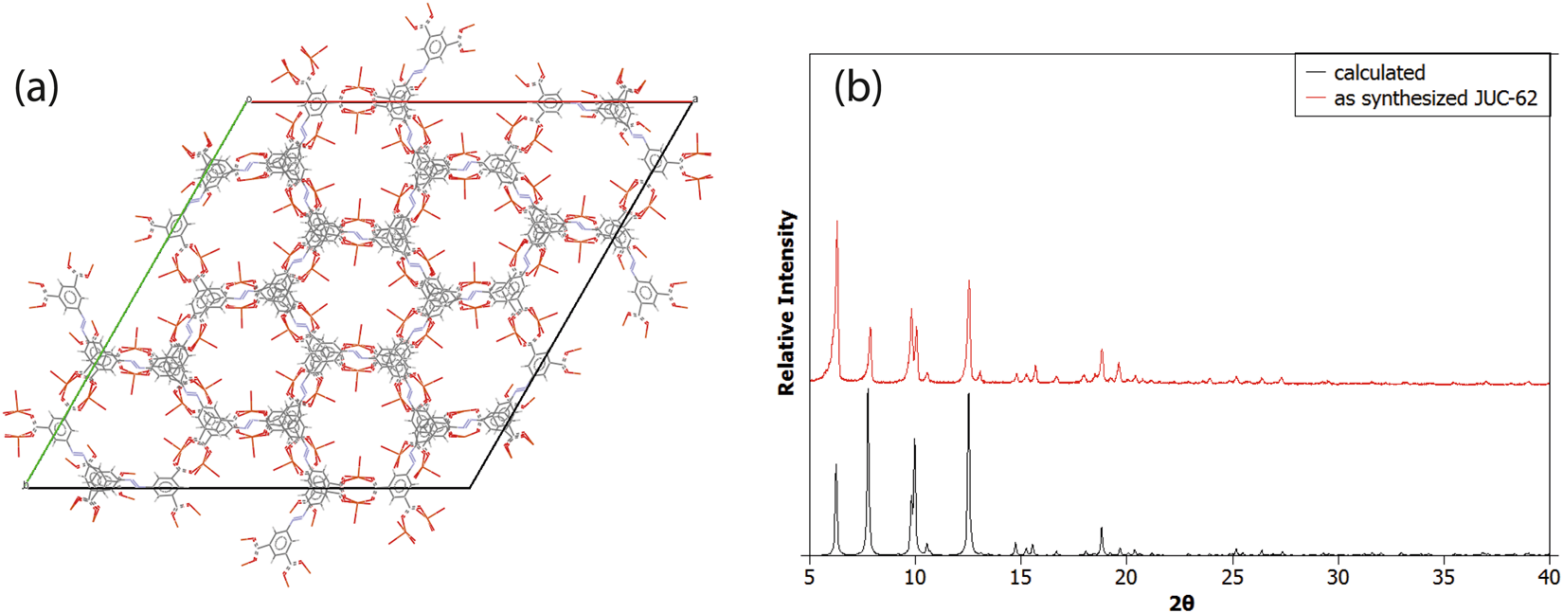
The structure of JUC-62 along its c axis (**a**) and its PXRD pattern of the as-synthesized and calculated JUC-62 (**b**).

JUC-62 is an NbO-type MOF with hexagonal channel built from copper paddlewheel and azobenzene tetracarboxylate ligand. Its hexagonal channel along its *c* axis as can be seen in Fig. 1(a). It was synthesized according to the previous literature^10^. Reaction between copper nitrate trihydrate and 3,3′-5,5′-azobenzene tetracarboxylic acid in a mixture of dimethylformamide (DMF), ethanol (EtOH) and water (5:3:1) at 60 °C for 2 days resulted in green block-shaped crystals. The obtained crystal was then characterized by powder X-Ray diffraction (PXRD) and the result is presented in Fig. 1(b).

As can be seen, both simulated pattern (CCDC: 666395) and the as-synthesized PXRD from JUC-62 matches very well indicating the right crystal was obtained. A difference observed in terms of relative intensity from the as-synthesized crystal which may indicate difference crystal orientation that occurred during crystal growth.

**Figure 2:**
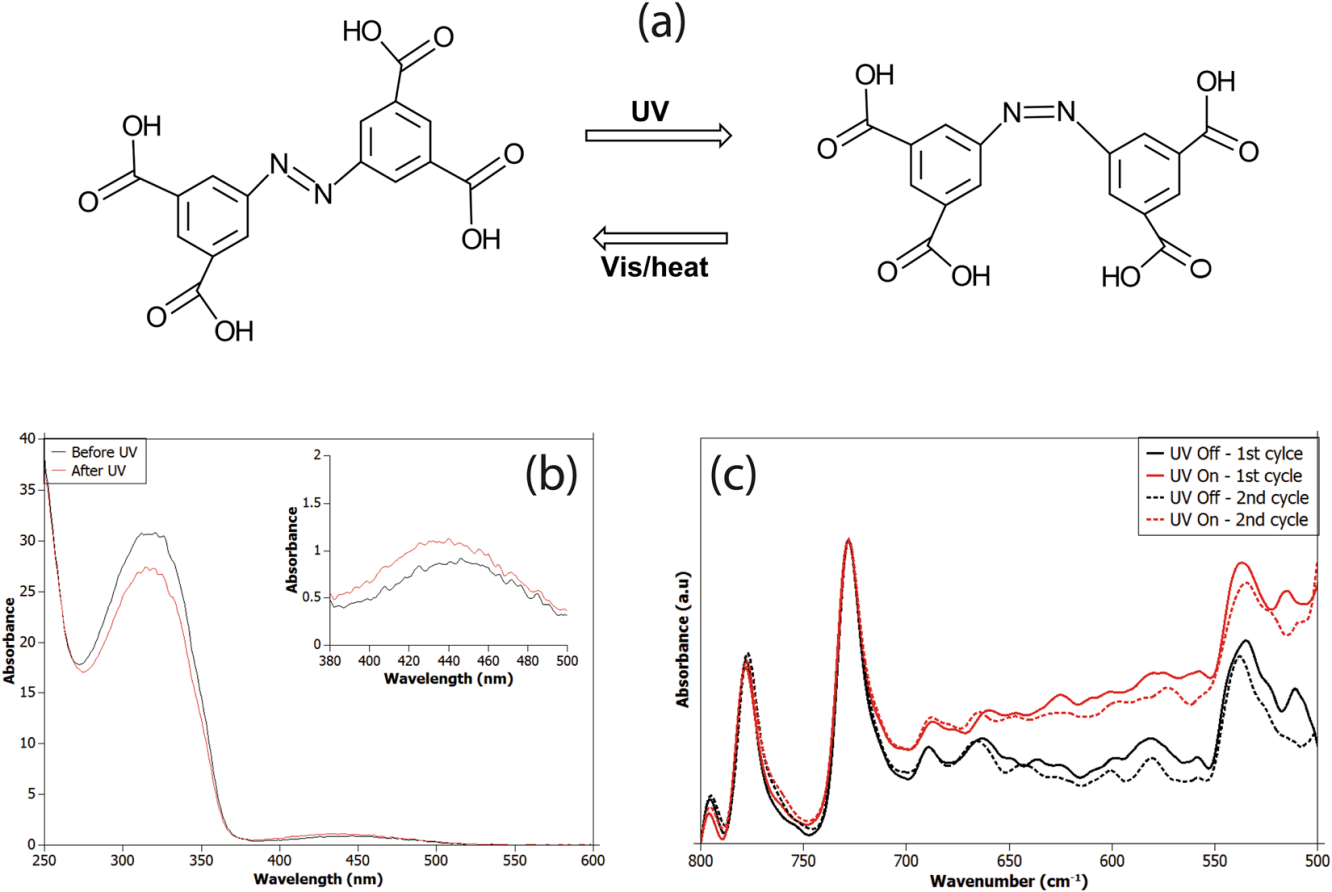
Isomerization visualization of the free ligand (**a**), its corresponding UV-Vis spectrum (**b**) and FTIR spectrum of JUC-62 (**c**).

Before conducting photo-switching study on JUC-62, we firstly investigated the photo-isomerization of the free ligand by recording its UV-Visible spectrum. ATR-FTIR spectrum of JUC-62 was also recorded before and after UV light irradiation. Both of the results are presented in Fig. 2.

It is well-known that azobenzene compounds can undergo photo-isomerization from its thermodynamically-stable *trans*-state to *cis*-state under UV light irradiation. A visualization of the photo-isomerization of the free ligand used in JUC-62 is depicted in Fig. 2(a). Such photo-isomerization was then proven by recording the UV-vis spectrum of the free ligand before and after UV irradiation. The spectrum is given in Fig. 2(a). From the spectrum, it can be seen that there are two characteristic azobenzene absorbance peaks observed from the free ligand: around 320 nm (π-π*) and 440 nm (n-π*). As can be seen, irradiation of the free ligand with UV light decreases the intensity of the absorbance peak at 320 nm. Meanwhile, an increase in the absorbance band at 440 nm was observed after the free ligand was irradiated with UV light. This then indicates the isomerization of the ligand occurs after UV light irradiation^11^. However, a remarkable difference in JUC-62 UV-Vis spectrum both in its pristine and UV-light-illuminated condition could not be clearly observed. Irradiation of JUC-62 with UV light under certain length of time did not give any significant change in terms of UV-vis spectra. This might be caused by the instantaneous change of the framework after the UV light was switched off and the time window between switching off the UV light and measurement was not short enough to maintain the framework to retain its structure under measurement.

Although any change in UV-vis spectrum for the JUC-62 could not be observed, a remarkable difference was observed in the FTIR spectrum of the JUC-62 crystal before and after UV light irradiation since the time window for this experiment was relatively short. As previously investigated, azobenzene localized bending could be observed in the region between 500–700 cm^−1^ in the FTIR spectrum. As can be seen from Fig. 2(c), the absorbance from 500–700 cm^−1^ region significantly increased when the sample was irradiated with UV light. The absorbance could go down to its initial state once the UV light was switched off. This phenomenon is attributed to the bending mode of C-C-C and C-C-N bonding in azobenzene network^1,8^.

**Figure 3:**
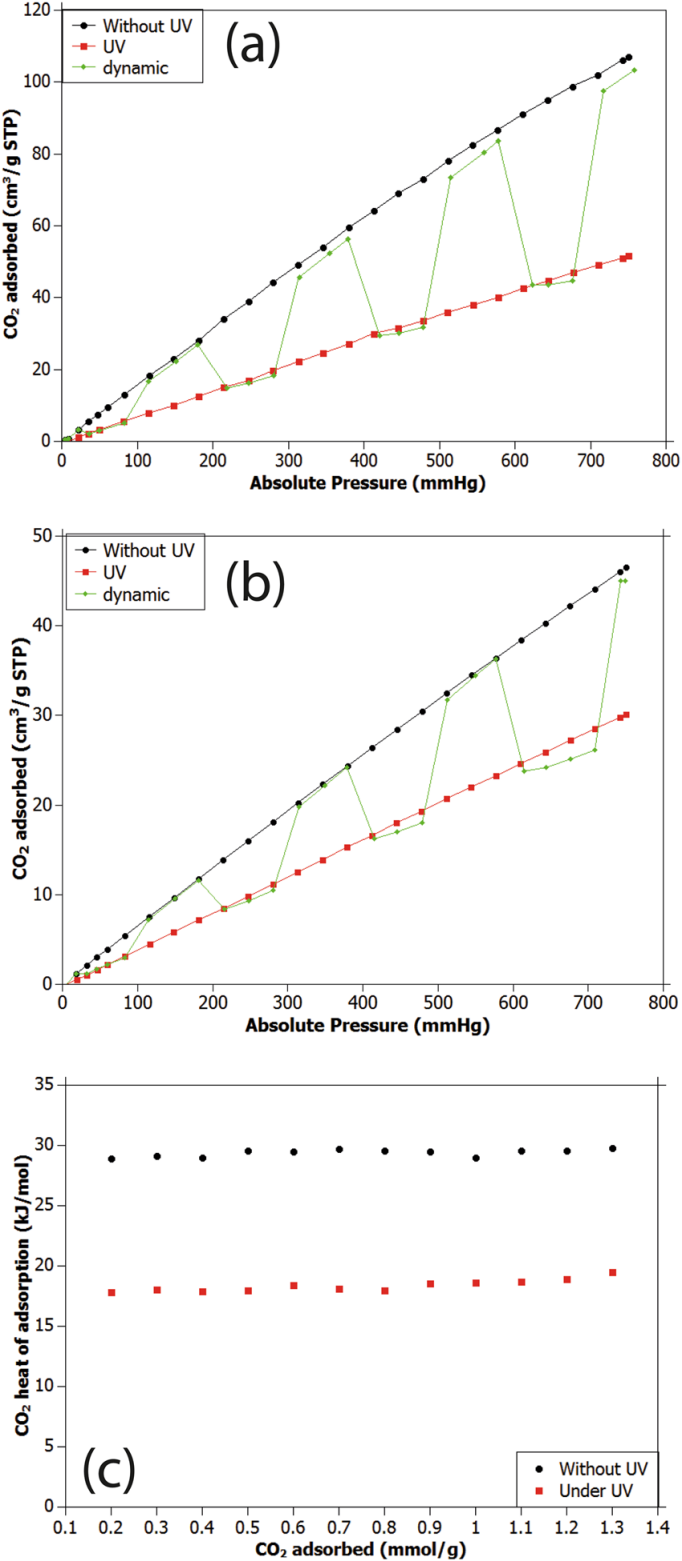
CO_2_ adsorption of JUC-62 in static and dynamic condition at (**a**) 273 K and (**b**) 298 K and JUC-62 CO_2_ heat of adsorption (**c**).

Since photo-responsive behaviour was exhibited by the JUC-62′s ligand and JUC-62 crystal FTIR spectrum had remarkable difference after UV light-irradiation, we then investigated the potentiality of JUC-62 as a light-responsive MOF for CO_2_ capture. In this study, we used two different temperatures: 273 K and 298 K. The results are presented in Fig. 3(a and b), respectively.

As can be seen, the CO_2_ adsorption capacity of JUC-62 at 750 mmHg was found to be around 102 cm^3^/g (STP) at 273 K. Meanwhile at 298 K, the CO_2_ adsorption capacity of JUC-62 was around 46 cm^3^/g (STP). This phenomenon is expected since higher temperature usually leads to lower adsorption capacity.

Interestingly, a decrease in CO_2_ adsorption capacity was not solely observed as the temperature was increased. As can be seen, in static condition with continuous UV light irradiation, JUC-62 liberated adsorbed CO_2_ resulting in reduction of CO_2_ adsorption capacity. We observed that at 750 mmHg and 273 K, JUC-62 adsorbed about 50% less CO_2_ when it was being illuminated by UV light. The amount of CO_2_ adsorbed at this condition was found to be around 52 cm^3^/g (STP). The same phenomenon could also be observed when the temperature was increased to 298 K. At this temperature, JUC-62 adsorbed about 34% less CO_2_ when it was being illuminated with UV light resulting in around 30 cm^3^/g adsorption capacity at 750 mmHg.

Thanks to its different uptake in the presence or absence of UV light, we then investigated the CO_2_ adsorption of JUC-62 under dynamic condition when the UV light was periodically switched on and off. As can also be seen in Fig. 3(a and b), the dynamic behaviour of JUC-62 matches very well with its static condition. When the UV light was switched on, the amount of CO_2_ adsorbed inside the JUC-62 framework dropped instantaneously and it could effectively reach back to its initial condition when the UV light was switched off. It is true that we cannot completely eliminate the effect of temperature increase of the sample when the UV light was on. As can be seen from supporting information, our control experiment using as-received Mil-53 (Al) BASF with no photoresponsive ligand showed about 7% decrease at 750 mmHg of CO_2_ uptake when the UV light was on (Supplementary Figure S3). This strongly suggests that the temperature inside the sample tube actually increased slightly. Therefore, we should have also expected a similar value of CO_2_ adsorbed decrease if JUC-62 were not photo-responsive. However, the 34% decrease in CO_2_ uptake of JUC-62 at the same condition cannot be merely explained by temperature increase inside the sample tube. To be more convinced that this decrease of CO_2_ uptake is actually a material property of JUC-62, we also did a control experiment using a light-responsive PCN-250 which has been published before. As can be seen from the supporting information, PCN-250 showed a dynamic CO_2_ uptake which is similar with what we observed in JUC-62. Thus, we could safely infer that such a dynamic behaviour of CO_2_ uptake is actually a characteristic property of the JUC-62 framework thanks to its photo-responsive ligand.

As previously discussed, the dynamic behaviour of JUC-62 might be caused by the localized bending experienced by the ligand when the UV light was turned on^1,8^. This is because the ligand cannot undergo complete photo-isomerization when being irradiated with UV light as experienced in its free state because of the rigid JUC-62 framework. The advantage of this restricted bending is then the framework can then be instantaneously brought back to its initial condition when the UV light was turned off. As can be observed, once the UV light was turned off, the JUC-62 returned to its initial condition and thus fully restored its initial CO_2_ adsorption capacity^8^.

From the data obtained at 273 and 298 K and by using Clausius-Clapeyron fitting, the CO_2_ heat of adsorption of JUC-62 both in its pristine and UV-light-illuminated condition can also be calculated. In its pristine condition, the JUC-62 CO_2_ heat of adsorption was found to be around 29 kJ/mol. In contrast, under illumination of UV light, heat of adsorption of JUC-62 was lower and found to be around 17 kJ/mol. Since the value of CO_2_ heat of adsorption of JUC-62 in pristine condition was higher than under illuminated condition, the interaction between CO_2_ and JUC-62 framework is stronger than under the UV-illuminated condition. The weaker interaction between CO_2_ and JUC-62 framework under UV-light irradiation might then trigger the instantaneous release of CO_2_ when JUC-62 was irradiated with UV light, resulting in lower CO_2_ uptake.

Since JUC-62 also has a catenated structure that can improve its stability^10,12^, we are also interested to observe its stability when JUC-62 powder was stored without employing any special treatment such as under inert condition or stored in a glove box.

**Figure 4:**
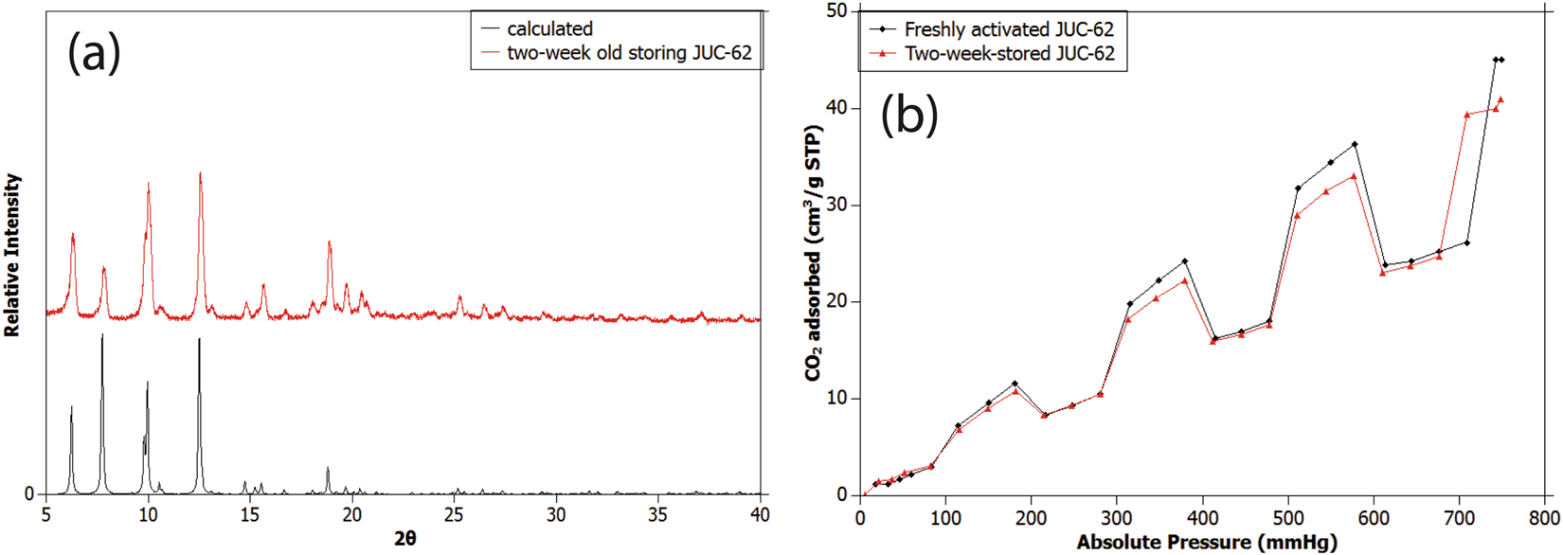
PXRD pattern of two-weeks old JUC-62 (**a**) and its dynamic CO_2_ adsorption (**b**).

As can be seen in Fig. 4(a), both calculated and PXRD pattern of JUC-62 still match very well indicating the integrity of the JUC-62 framework during the storing period. This was also then proven by performing dynamic CO_2_ adsorption of the two-week old JUC-62. Although a slight drop in CO_2_ adsorption capacity at 750 mmHg was observed from 46 cm^3^/g (STP) to be around 42 cm^3^/g (STP), it can be clearly seen that its photo-switching ability remains intact. We still observed about 34% drop in CO_2_ adsorbed at 750 mmHg for the two week old sample indicating a very good structural integrity of the framework during storage.

In conclusion, we have shown the applicability of JUC-62 beyond its hydrogen storage, namely as another candidate for generation-3 light-responsive MOF. Since its framework incorporates azobenzene moiety, JUC-62 exhibited lower CO_2_ uptake when it was irradiated with UV light. Interestingly, under dynamic condition when UV light was periodically switched on and off, the CO_2_ uptake of JUC-62 matched very well with its static condition, either with or without UV. Moreover, thanks to its catenated structure, its framework was found to be still intact when it was stored under ambient condition.

## Methods

### Synthesis of 3,3′-5,5′-azobenzene tetracarboxylic acid

3,3′-5,5′-azobenzene tetracarboxylic acid was synthesized according to the previous literature^13^. In a typical synthesis, 5 grams of 5-nitroisophthalic acid was dissolved in a solution containing 12.5 gram of NaOH in a 62.5 mL ultrapure water. The solution was stirred at 60 °C for 1 hour until a homogeneous yellowish slurry was obtained. Meanwhile, 25 gram of glucose was dissolved into 37.5 mL of ultrapure water and heated at 60 °C. The glucose solution was then added dropwise and slowly to the nitroisophthalic solution. After all of the glucose solution was added, the main solution turned dark brown. Afterwards the main solution cooled to room temperature without further stirring. This was then followed by overnight air bubbling of the solution. The solids obtained after this process were collected by filtering the solution under reduced pressure and then dissolved in 100 mL of ultrapure water. This was followed by acidification by using 2 M hydrochloric acid (HCl) and was further acidified using 37% HCl until pH = 1 to obtain an orange precipitate. The precipitate was then collected by filtration and washed with ultrapure water. The precipitate was then dried in a vacuum oven at 110 °C overnight.

### Synthesis of JUC-62

JUC-62 was synthesized according to the previous literature^9^. In a typical synthesis, 0.36 gram of copper nitrate trihydrate and 0.24 gram of 3,3′-5,5′-azobenzene tetracarboxylic acid was suspended in a mixture of DMF: EtOH: water (50 mL: 30 mL: 10 mL) in a glass vial. The solution was then ultrasonicated for about 15 minutes until a uniform suspension was obtained. Afterwards, 2 M nitric acid (HNO_3_) was added dropwise. Each drop of HNO_3_ was followed by stirring. This process was continued until a clear yellowish solution was obtained. This final solution was then put inside an oven heated at 60 °C for 2 days. After 2 days, green crystals were formed at the bottom of the glass vial. The powder was collected by filtration and washed with acetone. The powder was then kept in acetone for more than 2 days to exchange the solvent.

### JUC-62 CO2 dynamic photo-switching

The dynamic photo-switching and static CO_2_ uptake of JUC-62 was measured using 3 Flex Micromeritic instrument. Before each measurement, the sample was activated under vacuum at 110 °C overnight. Around 30 mg of sample was then put into a quartz tube (purchased from Micromeritic) during the measurement. An aluminium light-box designed according to the previous study^8^ was used to maximize the light exposure to the sample. Omnicure S1500 equipped with 365 nm filter was used as the high-intensity light source and the intensity was set at the maximum (around 3.8 W/cm^2^, measured using R2000 radiometer). During the measurement, the temperature was controlled by using Micromeritic ISO Controller filled with water or a mixture of water and ethylene glycol (for 0 °C measurement) as the coolant. CO_2_ adsorption was measured at low pressure between 0–750 mmHg.

For control experiment, PCN-250 was synthesized according to the previous publication^1,14^. Meanwhile, Mil-53(Al) was kindly provided by BASF and was used as-received without any treatment.

### Other characterizations

Purity of the ligand was evaluated by using Bruker nuclear magnetic resonance (NMR) spectroscopy. Proton and carbon NMR spectroscopy was recorded using 400 and 101 MHz instrument, respectively, and using d6-DMSO as the solvent.

PXRD spectrum was collected using PANalytical X-Ray Diffractometer Instrument. The power of the instrument was set to 40 kV and 20 mA. Copper Kα was used as the X-ray source.

FTIR spectrum was collected using Perkin-Elmer Spectrum 100 ATR-FTIR Spectrometer. For after-UV light experiment, the sample was first irradiated with the Omnicure S1500 for about 5 minutes. This was then quickly followed by pressing to the ATR-FTIR equipment and FTIR spectrum was collected.

UV-Vis spectrum of the free ligand was collected using Thermo Scientific NanoDrop 2000 Spectrophotometer. The ligand was dissolved in dimethylsulfoxide (DMSO) before the measurement. The ligand solution was then irradiated using a 365 nm UV light LED purchased from Thorlab (3.65 V; 1.1 Amp) for 40 min before the after-UV measurement took place.

CCDC number for JUC-62 is 666395. Figure 1(a) in the manuscript is generated by using Mercury software.

## Electronic supplementary material

Supplementary Information
