# Supplementary material for: Dynamic photo-switching in light-responsive JUC-62 for CO2capture
Source: Sci Rep. 2017 Oct 17;7:13355. doi: 10.1038/s41598-017-13536-4 (PMC5645403; doi:10.1038/s41598-017-13536-4)
Supplement: Supplementary file 1 — Supplementary Information [file 41598_2017_13536_MOESM1_ESM.pdf]

Supplementary Information

Dynamic photo-switching in light-responsive JUC-62 for CO_2 capture

Nicholaus Prasetya[1] and Bradley Ladewig[1,*]

[1]Barrer Centre, Chemical Engineering Department, Imperial College London, Exhibition Road, London SW7 2AZ, United Kingdom

[*]b.ladewig@imperial.ac.uk

Contents

1.	H-NMR spectrum of 3,3’-5,5’-azobenzene tetracarboxylic acid	S2
2.	C-NMR spectrum of 3,3-5,5’-azobenzene tetracarboxylic acid	S3
3.	CO_2 adsorption control experiment	S3

1. **H-NMR Spectrum of 3,3'-5,5'-azobenzene tetracarboxylic acid**

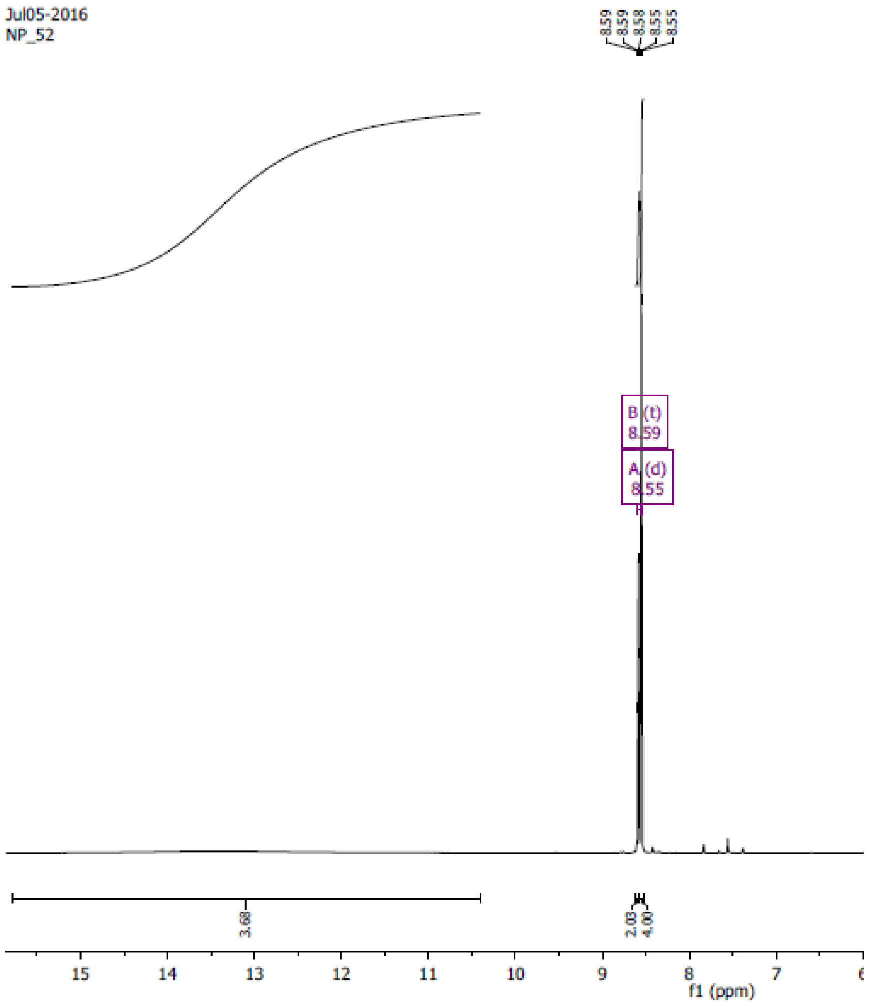

(a)

Supplementary Figure S 1. H-NMR spectrum of 3,3'-5,5'-azobenzene tetracarboxylic acid (d6-DMSO) (^{1}H NMR (400 MHz, DMSO) δ 8.61-8.57 (t, *J* = 1.6 Hz, 2H), 8.56-8.54 (d, *J* = 1.6 Hz, 4H))

2. C-NMR spectrum of 3,3-5,5'-azobenzene tetracarboxylic acid

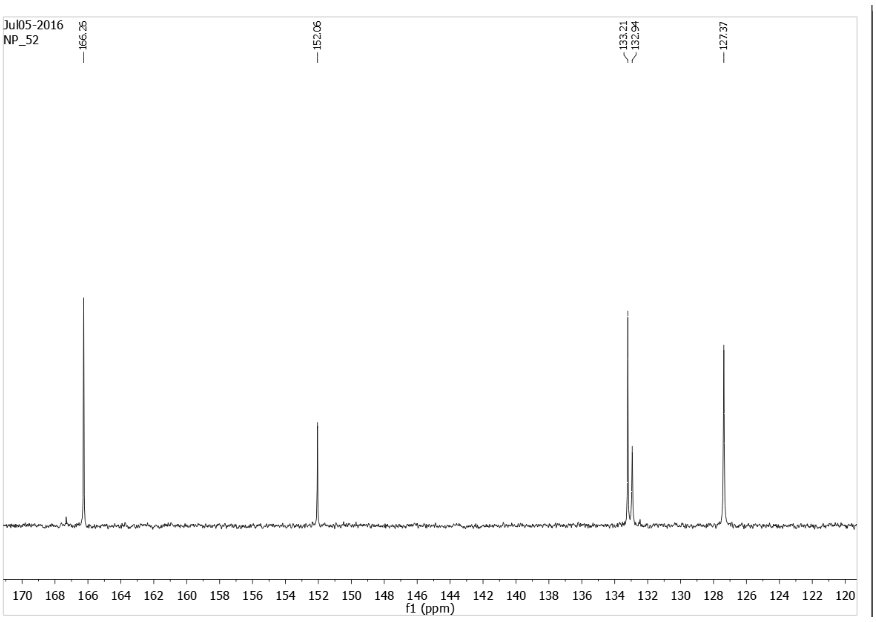

Supplementary Figure S 2. C-NMR Spectrum of 3,3'-5,5'-azobenzene tetracarboxylic acid (^{13}C NMR (101 MHz, DMSO) δ 166.26, 152.06, 133.21, 132.94, 127.37)

3. CO_2 adsorption control experiment

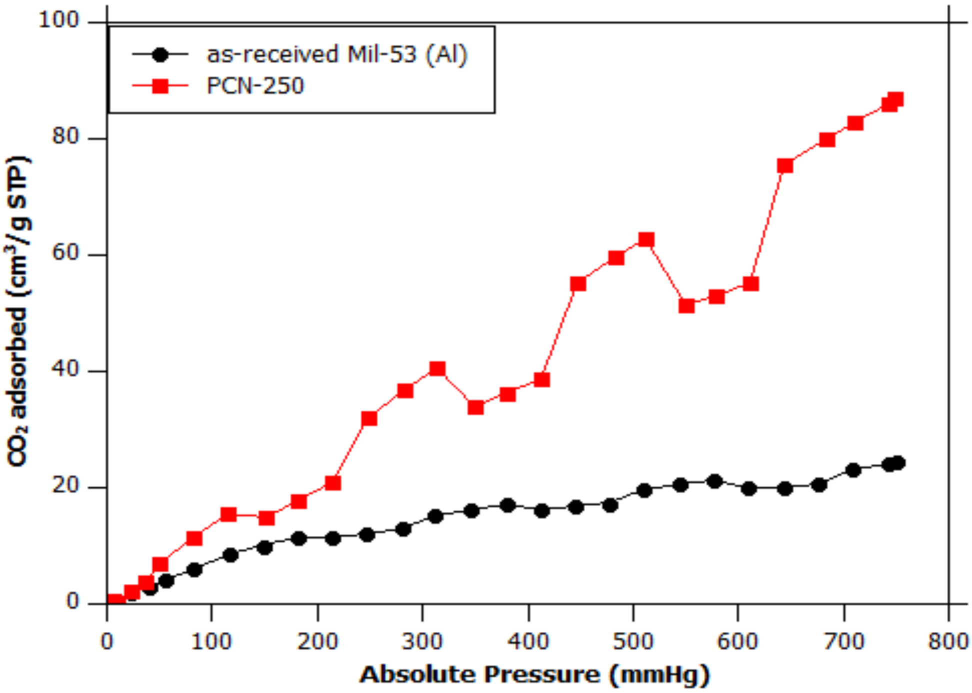

Supplementary Figure S 3. CO_2 adsorption dynamic photoswitching experiment using as-received Mil-53(Al) BASF and PCN-250
